# Effects of Locomotion in Auditory Cortex Are Not Mediated by the VIP Network

**DOI:** 10.3389/fncir.2021.618881

**Published:** 2021-04-07

**Authors:** Iryna Yavorska, Michael Wehr

**Affiliations:** Department of Psychology, Institute of Neuroscience, University of Oregon, Eugene, OR, United States

**Keywords:** VIP neuron, running, disinhibition, auditory cortex, interneurons, state modulation, sound encoding

## Abstract

Movement has a prominent impact on activity in sensory cortex, but has opposing effects on visual and auditory cortex. Both cortical areas feature a vasoactive intestinal peptide-expressing (VIP) disinhibitory circuit, which in visual cortex contributes to the effect of running. In auditory cortex, however, the role of VIP circuitry in running effects remains poorly understood. Running and optogenetic VIP activation are known to differentially modulate sound-evoked activity in auditory cortex, but it is unknown how these effects vary across cortical layers, and whether laminar differences in the roles of VIP circuitry could contribute to the substantial diversity that has been observed in the effects of both movement and VIP activation. Here we asked whether VIP neurons contribute to the effects of running, across the layers of auditory cortex. We found that both running and optogenetic activation of VIP neurons produced diverse changes in the firing rates of auditory cortical neurons, but with distinct effects on spontaneous and evoked activity and with different patterns across cortical layers. On average, running increased spontaneous firing rates but decreased evoked firing rates, resulting in a reduction of the neuronal encoding of sound. This reduction in sound encoding was observed in all cortical layers, but was most pronounced in layer 2/3. In contrast, VIP activation increased both spontaneous and evoked firing rates, and had no net population-wide effect on sound encoding, but strongly suppressed sound encoding in layer 4 narrow-spiking neurons. These results suggest that VIP activation and running act independently, which we then tested by comparing the arithmetic sum of the two effects measured separately to the actual combined effect of running and VIP activation, which were closely matched. We conclude that the effects of locomotion in auditory cortex are not mediated by the VIP network.

## Introduction

Movement has complex effects on activity in auditory cortex, and involves multiple pathways. A variety of movements, such as blinking, grooming, and running, produce widespread suppression of sound-evoked and spontaneous spiking activity, which reduces the amount of stimulus information as well as coding efficiency (Nelson et al., 2013; Schneider et al., 2014; Bigelow et al., 2019). A key source of movement information is secondary motor cortex (M2), which projects to both excitatory pyramidal neurons (PNs) and to parvalbumin-expressing (PV) inhibitory interneurons in auditory cortex, producing diverse effects that overall yield a net suppression of PNs (Schneider et al., 2014). Other (non-PV) inhibitory interneurons also show movement-related increases in activity, but since they are not targeted by M2 projections (Schneider et al., 2014), they are likely affected by one of at least two other pathways for movement information. Locomotion suppresses activity in layer 2/3 of auditory cortex by decreasing thalamic and intracortical synaptic drive (Zhou et al., 2014). In addition, locomotion recruits cholinergic signaling from the basal forebrain, which targets both excitatory and inhibitory cortical neurons (Hangya et al., 2015; Nelson and Mooney, 2016). How these multiple pathways interact, and how they account for the diversity of movement effects across individual cells, remains poorly understood.

Locomotion has the opposite effect in visual cortex, where it *increases* the gain of visually evoked responses, without affecting tuning for visual features (Niell and Stryker, 2008). It remains unclear whether the mechanisms by which locomotion modulates activity in visual and auditory cortex are similar, partially overlapping, or completely distinct. Vasoactive intestinal peptide-expressing (VIP) inhibitory interneurons have emerged as key players in a disinhibitory circuit motif by which locomotion can increase spiking activity in PNs. VIP neurons comprise a small fraction (10–15%) of all inhibitory neurons, corresponding to only 1–2% of all cortical cells (Gonchar et al., 2007; Pfeffer et al., 2013). They are found in all cortical layers, with the highest density in layer 2/3 (Xu et al., 2010). In visual cortex, locomotion activates VIP neurons via nicotinic signaling from the basal forebrain, and VIP neurons in turn inhibit somatostatin-expressing (SOM) interneurons to cause a net increase in PN spiking (Fu et al., 2014). A similar disinhibitory circuit has also been reported in barrel cortex during locomotion, where VIP neurons receive input from motor cortex, resulting in disinhibition of PNs during whisking (Lee et al., 2013). In auditory cortex, although VIP circuitry appears broadly similar to that in visual cortex (Pfeffer et al., 2013; Nelson and Mooney, 2016), it remains unknown whether VIP neurons are activated by locomotion or not. The VIP disinhibitory circuit in auditory cortex is recruited during an auditory discrimination task; rewards activate VIP neurons for an extended period of time, whereas punishments activate VIP neurons only transiently (Pfeffer et al., 2013; Pi et al., 2013). VIP inhibition of SOM and some PV neurons results in a net increase of tone responses in PNs (Pi et al., 2013; Bigelow et al., 2019). The basal forebrain projects to all major types of auditory cortical neurons, including VIP neurons, and either locomotion or activation of cholinergic basal forebrain axons depolarizes auditory cortical neurons (Nelson and Mooney, 2016). The fact that VIP activation facilitates PNs in both auditory and visual cortex, but that locomotion has opposing effects in auditory and visual cortex, suggests that the mechanisms underlying movement effects in auditory cortex are more complex than the locomotion-VIP disinhibitory circuit in visual cortex. Although both M2 and the basal forebrain convey movement-related signals to auditory cortex, the extent of convergence of this input across cortical layers, and the timescale of its strongest recruitment and influence on auditory processing, remain unknown.

The functional roles of VIP neurons in auditory cortical processing also remain unclear. Characterization of VIP cells has been mostly restricted to upper layers or to a small number of patched cells in deeper layers (Lee et al., 2013; Pfeffer et al., 2013; Pi et al., 2013; Fu et al., 2014; Nelson and Mooney, 2016). Broad activation of VIP cells increases firing rates in auditory cortex without any corresponding increase in the amount of stimulus information conveyed, resulting in a net decrease in coding efficiency (Bigelow et al., 2019). Thus VIP activation and running have opposing effects on evoked firing rates in auditory cortex (a net increase vs. a net decrease). Indeed, the combined effects of running and VIP activation are well-predicted by the additive sum of the effects of running and VIP activation measured separately, suggesting that the two effects act through independent pathways (Bigelow et al., 2019). However, both effects are quite diverse at the single-cell level, and it is unknown whether they differ across cortical layers. Moreover, it seems likely that VIP cells in different layers play distinct roles in auditory processing. About 60% of VIP neurons are located in layer 2/3, and the fact that they maintain their dendrites in superficial layers with local axonal projections only within their own layer or to layer 5a suggests that these L2/3 VIP cells are those that have been implicated in the disinhibitory motif (Fu et al., 2014). The other 40% in layers 4 and 5 seem to exhibit a different connectivity pattern, the function of which is still not well understood. The differences in dendritic and axonal projection patterns between VIP cells in the superficial and deep layers suggest that their roles might not be restricted only to state modulation via a disinhibitory circuit. Laminar differences in the roles of VIP circuitry could thus contribute to the substantial diversity that has been observed in the effects of both movement and VIP activation (Nelson and Mooney, 2016; Bigelow et al., 2019). Resolving these issues requires recording from auditory cortex with high laminar precision during both running and VIP activation.

We therefore recorded from auditory cortical neurons in awake mice that expressed ChR2 in VIP-positive neurons, during natural changes in arousal and locomotion. We used linear silicon probe arrays and current-source density analysis to obtain precise cortical depths of recorded neurons. Consistent with previous work, we found that activating VIP cells produced an overall facilitation of cortical neurons, but the effects were quite diverse across the population, with 44% of neurons showing an increase in spontaneous firing and 27% showing a decrease. Facilitated and suppressed neurons were found in all cortical layers, with a strong and specific reduction in the encoding of sound in layer 4 narrow-spiking neurons. Locomotion also had diverse and layer-dependent effects on neuronal activity, which showed a different pattern of facilitation and suppression than VIP activation. Overall, running led to an increase in spontaneous activity but a suppression of evoked activity, with the strongest reduction in the encoding of sound in layer 2/3. Changes in neuronal firing when the animal was running during VIP activation trials were well-predicted by the sum of VIP activation and running effects measured separately. Taken together, these results suggest that the modulatory effects of running in auditory cortex are not mediated by the VIP inhibitory network.

## Results

**FIGURE 1:**
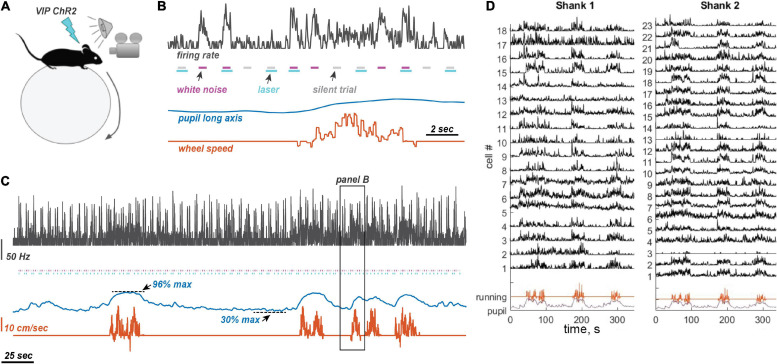
Experimental design and measurements. **(A)** Experimental setting. Awake mice were allowed to run on a ball. Sounds were presented randomly interleaved with laser illumination. Pupil size was measured on the contralateral side from the neural recording site (left auditory cortex and right pupil). **(B)** Stimulus presentation. Laser pulses were presented with and without 80 dB WN bursts, randomly interleaved, with a 1 s inter-stimulus interval. When presented, the laser pulse began 50 ms before the start of the sound and ended 150 ms after sound offset. **(C)** Example traces of neuronal firing rate (25 ms time bins, Gaussian convolution smoothing with σ = 50 ms), pupil size, and running speed. Color coding of traces is the same as in panel **(B)**. Animals frequently oscillated between low and high arousal states. **(D)** Example traces from 41 simultaneously recorded neurons using a two-shank linear silicon probe, showing typical modulation by running.

We recorded from excitatory and inhibitory neurons in auditory cortex in awake head-fixed VIP-ChR2 mice (*N* = 10 mice; included in analysis of VIP activation and locomotion) and PV-ChR2 mice (*N* = 6 mice; included in analysis of locomotion effects only) that were allowed to run on a spherical ball (Figure 1; *N* = 16 mice in total). In addition to locomotion, we also recorded pupil size with an IR camera to monitor the state of arousal during recordings. As expected, running events only occurred during states of high arousal (above 60% of maximum recorded pupil size, Supplementary Figure 1). To examine the influence of running, we sorted stimulus presentations (trials) by running speed. We classified trials on which the average speed was above 5% of maximum speed as running trials. Only recordings with at least 7 or more running trials were included in analysis. On average, mice were running on 13 ± 2% of all trials. Average speed on running trials was 6.96 ± 0.16 cm/s, and on sitting trials was 0.02 ± 0.0014 cm/s.

### Effects of Running

**FIGURE 2:**
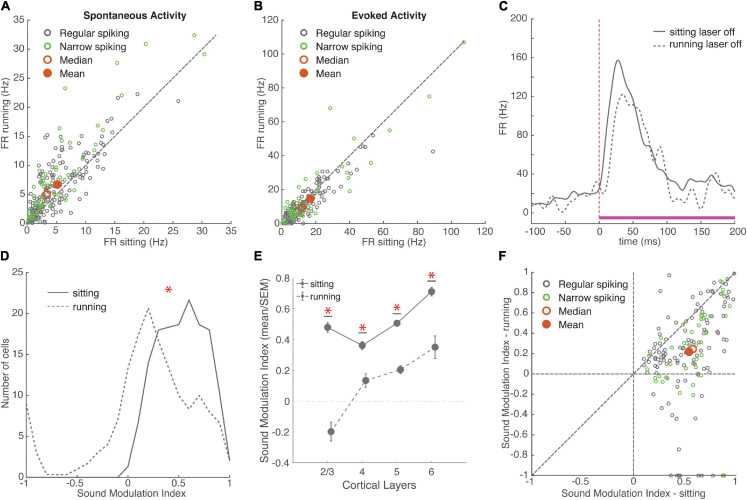
Running had variable effects on neural activity, overall increasing spontaneous firing rates and reducing the encoding of sounds. **(A)** Spontaneous firing rate during sitting and running trials. Green: narrow-spiking neurons, grey: regular-spiking neurons. Red filled circle: population mean, red unfilled circle: median. Running FR: 6.50 ± 0.38 Hz, sitting FR: 4.87 ± 0.32 Hz, mean ± SEM, *N* = 235 cells, signed-rank *p* = 10^–14^, effect size *r* = 0.35. Dashed line is unity in all figures. **(B)** Onset response firing rate evoked by white noise stimulus (0–100 ms) during sitting and running trials (without baseline subtraction). Running FR: 13.97 ± 1.09, sitting FR: 15.81 ± 1.18, *N* = 177 cells, signed-rank *p* = 10^–5^, effect size *r* = 0.22. **(C)** Example response to a white noise stimulus in two behavioral conditions. Mean response during sitting trials plotted with solid grey line, mean response during running trials plotted with dashed grey line. White noise stimulus is shown in magenta with a dashed line indicating the onset of the stimulus. **(D)** Distributions of sound modulation indices during sitting (solid line) and running (dashed line). Sitting: 0.54 ± 0.02, running: 0.23 ± 0.04, *N* = 154 cells, signed-rank *p* = 10^–19^, effect size *r* = 0.52. **(E)** Mean and SEM of sound modulation indices across cortical layers in sitting and running conditions (means ± SEM, L2/3 sitting = 0.48 ± 0.03, running = –0.20 ± 0.06, *N* = 10; L4 sitting = 0.36 ± 0.03, running = 0.13 ± 0.04, *N* = 19; L5 sitting = 0.51 ± 0.01, running = 0.20 ± 0.03, *N* = 58; L6 sitting = 0.71 ± 0.03, running = 0.35 ± 0.07, *N* = 14). **(F)** Comparison of sound modulation index on sitting trials vs. running trials for each cell. *indicates statistical significance.

Although running produced diverse effects on spontaneous and evoked activity, we found that most auditory neurons increased their spontaneous firing rate during periods of locomotion (Figure 2A). We measured spontaneous firing rate as the mean firing during interleaved blank (silent) stimuli, which matched the durations and presentation intervals of sound stimuli. We classified cells based on spike waveform into narrow-spiking (NS) and regular-spiking (RS) cells. The running-induced increase in spontaneous firing was higher for NS neurons than for RS neurons (Figure 2A; Mean ± SEM FR change: RS = +1.27 ± 0.15 Hz, NS +2.70 ± 0.30 Hz, rank-sum *p* = 0.002, effect size *r* = 0.20), but similar across cortical layers (χ^2^ (3,137) = 0.69, *p* = 0.87). Auditory neurons show both onset and offset responses to sound, and we first examined changes in onset responses (measured in a 100 ms window following stimulus onset). Onset responses showed a modest but significant decrease during running (Figure 2B). This overall decrease in evoked firing rate was also true for offset responses (measured in a 100 ms window following stimulus offset, Mean ± SEM Offset response: running 12.06 ± 0.78 Hz, sitting 12.85 ± 0.78 Hz, *p* = 0.01, *N* = 206 cells, effect size *r* = 0.13, Supplementary Figure 2), which suggests that running similarly affects multiple aspects of sound processing. Running effects on evoked firing rates were similar between RS and NS cells (Mean ± SEM FS change: RS = −2.20 ± 0.31 Hz, NS = −0.83 ± 0.77 Hz, *p* = 0.48) as well as across all cortical layers (χ^2^ (3, 120) = 3.27, *p* = 0.23). Running significantly increased response latency in both RS cells (*p* = 0.014, sign-rank, latency difference: 4.9 ± 1.2 ms, *r* = 0.17) and FS cells (*p* = 0.014, latency difference: 9.3 ± 1.7 ms, *r* = 0.27; Supplementary Figure 3). Despite these net effects at the population level, the effects of running on individual cells were quite heterogeneous (Figures 2A,B).

To examine how these diverse running effects on spontaneous and evoked firing rates impacted sound encoding, we calculated a sound modulation index (MI) for each neuron separately on running and sitting trials. MI measures the net difference between evoked and spontaneous firing, and ranges from −1 to +1 (see section “Materials and Methods”). We found that the sound MI was significantly higher when the animals were sitting than when running, indicating that running reduces the effect of sound on firing (Figures 2D–F). Because there were far fewer running than sitting trials, we next compared sound MI using matched numbers of sitting and running trials, using a randomly sampled subset of sitting trials. We repeated this process 100 times to obtain a distribution of randomly sampled means and signed-rank *p*-values. The MI was significantly higher for sitting in each and every sample (Mean ± SEM running MI = 0.23 ± 0.04; matched sitting samples: MI = 0.50 ± 0.03, range: 0.42–0.55, mean *p* = 10^–12^, effect size *r* = 0.63 ± 0.004). The decrease in MI during running was observed across all cortical layers, but was most pronounced in layer 2/3 (Figure 2E; χ^2^ (3, 97) = 16.97, *p* = 0.0007). This decreased MI during running was similar for both narrow-spiking and regular-spiking neurons. Although previous work has shown that running suppresses auditory cortex by recruiting PV inhibitory neurons (Nelson et al., 2013), we observed a similar decrease in sound MI in both regular and narrow-spiking neurons (Figure 2F; Mean ± SEM change in sound MI: RS = −0.32 ± 0.04, NS = −0.30 ± 0.05, *p* = 0.5682), with the largest reduction in layer 2/3. This indicates that running decreases the responsiveness to sound in both excitatory and inhibitory neurons.

To further examine the strength and time course by which running modulated population activity, we recorded spontaneous activity during prolonged periods of silence, while the animal’s behavioral state changed naturally without external cues. Because arousal and locomotion may have non-linear effects on neural activity (such as an inverted-U relationship), we used the distance correlation (Székely et al., 2007), which captures both linear and non-linear relationships. We measured the relationship between running speed and neural activity by computing the distance correlation jointly between running speed and the firing rates of all simultaneously recorded neurons, in 100 ms time bins during each session. Running speed was significantly correlated with population activity (distance correlation = 0.36 ± 0.02, permutation test *p* = 0, *N* = 67 simultaneously recorded populations in 12 mice), confirming that running strongly modulates firing in auditory cortex. To test the time scale of this modulation, we computed distance correlations while binning firing rate in time bins ranging from 50 ms to 12.8 s. We found that distance correlation values were significantly positive across a wide range of time scale (50 ms to 3.2 s), with a peak at 0.4 s (distance correlation 0.42 ± 0.02, Supplementary Figure 3). This suggests that running modulates neural firing rate on a timescale of approximately half a second.

### VIP Modulation

**FIGURE 3:**
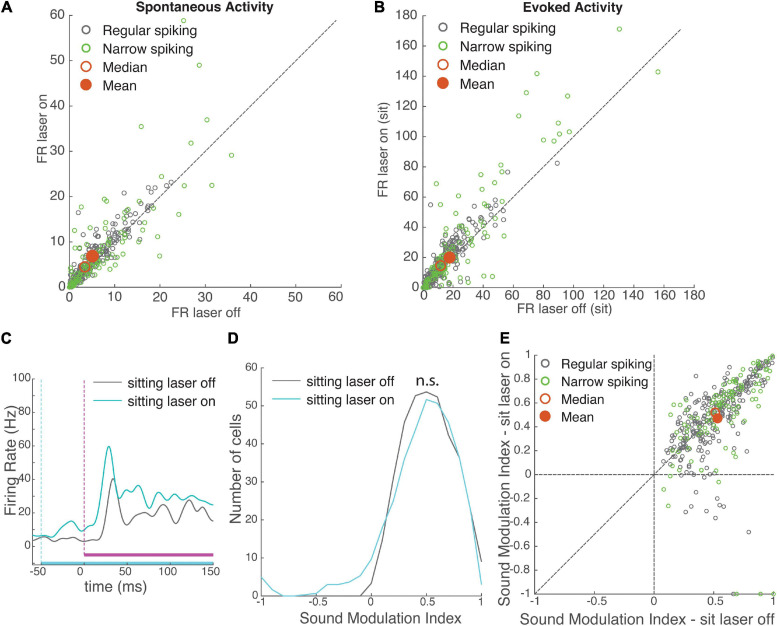
Effects of VIP activation on auditory cortical activity. VIP activation increased both spontaneous and evoked firing rates, with no net effect on modulation by sound. **(A)** Spontaneous firing rate of recorded neurons (*N* = 372) during laser-off and laser-on trials. Green: narrow-spiking neurons, grey: regular-spiking neurons. Red filled circle: population mean, red unfilled circle: median. **(B)** Onset response firing rate of recorded neurons (*N* = 372) to a white noise stimulus (0–100 ms post-stimulus onset) during laser-on and laser-off trials. **(C)** Mean response of an example neuron to a white noise stimulus during laser-off (grey) and laser-on (cyan) trials, while the mouse was sitting. White noise is depicted in magenta (vertical dashed line shows onset), laser is depicted in cyan (vertical dashed line shows onset). **(D)** Distributions of sound modulation indices while the mouse was sitting with (cyan) and without (grey) VIP activation. VIP activation had no net effect on sound modulation index (sound MI laser-off = 0.53 ± 0.01, laser-on = 0.47 ± 0.02, rank-sum *p* = 0.12, *N* = 372 cells). **(E)** Comparison of sound modulation index in sitting laser-off vs. laser-on conditions for each cell (*N* = 372).

To examine the effect of VIP cells on the activity of surrounding auditory neurons in our VIP-ChR2 mice, we embedded white noise or silent trials in a laser pulse, and compared laser-on to laser-off trials (Figure 1B). To disentangle behavioral and VIP modulation, we first compared laser-on and laser-off trials only during stationary periods. Similarly to locomotion, activating VIP cells produced diverse facilitation and suppression effects on the firing rates of surrounding cortical neurons. Overall, activating VIP neurons significantly increased spontaneous and evoked activity in two-thirds of auditory neurons (Figures 3A,B, spontaneous effect size *r* = 0.26, evoked *r* = 0.32), consistent with previous findings that VIP neurons form disinhibitory circuits that produce a net increase in cortical activity (Pfeffer et al., 2013; Bigelow et al., 2019). This significant net increase in activity was observed in both regular-spiking and narrow-spiking neurons. The increase in evoked firing rates was similar for RS and NS cells (rank-sum *p* = 0.32, *N* = 267 RS cells and 105 NS cells), whereas the increase in spontaneous firing rates was greater for RS than for NS cells (*p* = 0.03, possibly because NS cells had significantly higher spontaneous rates to begin with, *p* = 0.01). Thus activation of VIP cells increased the firing of both excitatory and inhibitory neurons. Unlike running, which increased response latencies, VIP activation modestly decreased response latency in both RS cells (*p* = 0.02, sign-rank, latency difference: −2.4 ± 0.8 ms, *r* = −0.23) or FS cells (*p* = 0.07, latency difference: −4.7 ± 1.3 ms, *r* = −0.21; Supplementary Figure 3).

To understand how the activation of VIP cells impacted sound encoding, we compared the sound MI for each neuron when the sound was embedded in a laser pulse (sitting laser-on) to sound without a laser pulse (sitting laser-off), during stationary periods. Even though VIP activation strongly modulated spontaneous and evoked firing rates, unlike running it did not have an overall effect on sound modulation indices (Figures 3D,E; effect size *r* = 0.07, rank-sum *p* = 0.12).

**FIGURE 4:**
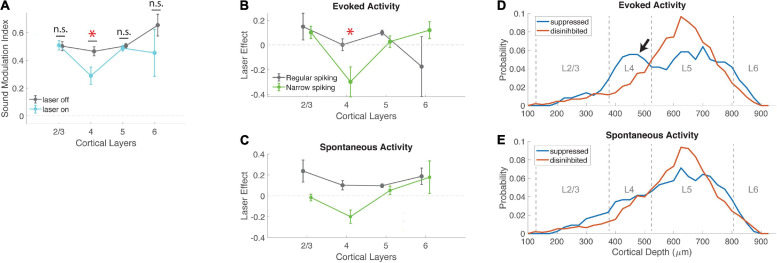
Effects of VIP activation are strongest in layer 4. **(A)** Mean sound modulation index during laser-on and laser-off trials, across cortical layers. VIP activation significantly suppressed modulation of neural activity by sound in layer 4, but not other layers (L2/3 laser-off 0.50 ± 0.03, laser-on 0.51 ± 0.03, *N* = 20; L4 laser-off 0.46 ± 0.03, laser-on 0.29 ± 0.06, *N* = 40; L5 laser-off 0.50 ± 0.02, laser-on 0.49 ± 0.02, *N* = 178; L6 laser-off 0.65 ± 0.08, laser-on 0.45 ± 0.17, *N* = 6; χ^2^ (3, 240) = 14.42, *p* = 0.0024; *post-hoc* signed-rank for L4 (MI laser-on vs. laser-off) *p* = 0.0014, *r* = 0.36). **(B)** The effect of VIP activation on sound modulation in layer 4 was driven by evoked activity in narrow-spiking neurons. Laser effect is the difference in evoked activity between laser-on and laser-off trials, normalized to each cell’s peak laser-off firing rate. Evoked activity in layer 4 narrow-spiking cells was significantly suppressed by VIP activation (NS χ^2^ (3, 73) = 10.06, *p* = 0.0141; *post-hoc* rank-sum for L4 laser effect <0: *p* = 0.0161; L4 NS vs. RS cells: *p* = 0.0230). **(C)** Laser effect for spontaneous activity in regular-spiking neurons was similar across all cortical layers, but for narrow-spiking cells was suppressed in L4 (NS: χ^2^ (3,73) = 8.8, *p* = 0.03). **(D)** Depth distribution of cells that were either suppressed or disinhibited by VIP activation, for evoked activity. Peak density of disinhibited cells was in layer 5; suppressed cells showed an additional peak in layer 4 (arrow). **(E)** Depth distributions of suppressed and disinhibited cells for spontaneous activity were similar to each other. Peak densities were in layer 5. *indicates statistical significance.

Vasoactive intestinal peptide-expressing neurons form distinct circuits in different cortical layers, and we therefore wondered whether the broadly increased firing rates without any change in sound encoding were uniform across layers, or varied with depth. Although most GABAergic targets of VIP neurons are located in the deep layers, and VIP cells are known to synapse onto different cellular compartments in excitatory and inhibitory neurons (Zhou et al., 2017), the functional outcome of these laminar and cell-type-specific connectivity differences are poorly understood. Surprisingly, we found that VIP activation had a dramatic impact on sound encoding only in layer 4 neurons (*p* = 0.0014; effect size *r* = 0.36, Figure 4A). Thus the lack of an overall effect on sound encoding when cells were pooled across all layers (Figures 3D,E; Bigelow et al., 2019) masks a specific suppression of sound encoding in layer 4.

Our measure of sound encoding (sound MI) is based on the contrast between evoked and spontaneous activity. We wondered whether the suppression of sound encoding in layer 4 was driven by a decrease in evoked activity, an increase in spontaneous activity, or both. We therefore examined laser effects on evoked and spontaneous activity separately (Figures 4B,C). We found that the reduction in sound MI in layer 4 was driven primarily by a strong and specific decrease in evoked responses in narrow-spiking neurons. In contrast, regular-spiking neurons were generally disinhibited by VIP activation for both spontaneous and evoked activity, with no layer-specific effects (Figures 4B,C and Supplementary Figure 4). Spontaneous activity in L4 NS cells was suppressed by VIP activation (Figure 4C), although this effect was in the wrong direction to account for the specific reduction in sound MI in layer 4. Because both sound MI and laser effects normalize the firing rate of individual neurons, we also verified that VIP suppression in layer 4 was not driven by neurons with low firing rates. This was not the case, since L4 had intermediate firing rates (between those of L2/3 and L5/6). In addition, the lower MI in L4 did not depend on choice of normalization. We repeated the analysis of Figures 4A–C with raw firing rates instead of MI or laser effects, and still observed a specific and significant suppression of evoked activity in L4 NS cells (NS χ^2^ (3,73) = 12.42, *p* = 0.0061, RS χ^2^ (3, 163) = 3.37, *p* = 0.34). How precisely is this effect limited to layer 4? To examine these effects by depth more closely, we compared the distribution of suppressed and facilitated cells across cortical depth (Figures 4D,E). Peak cell densities in our population were at depths of 600–700 μm, in layer 5, for both suppressed and facilitated cells. However, we also observed an excess of suppressed cells at a depth of 400–500 μm (in layer 4), for which VIP activation specifically suppressed evoked activity (arrow in Figure 4D). This shows that the suppressive effect of VIP activation on evoked activity in narrow-spiking cells (Figures 4A,B) was limited almost exclusively to layer 4. This selective suppression of sound modulation in the thalamorecipient layer suggests that the VIP network may specifically regulate bottom-up sensory information flow via modulation of sound responses in layer 4 inhibitory interneurons.

### Interaction Between Running and VIP Activation

Although both running and VIP activation produced diverse and distributed modulatory effects on neural activity, it remains unclear whether the VIP network is involved in endogenous modulation by running.

**FIGURE 5:**
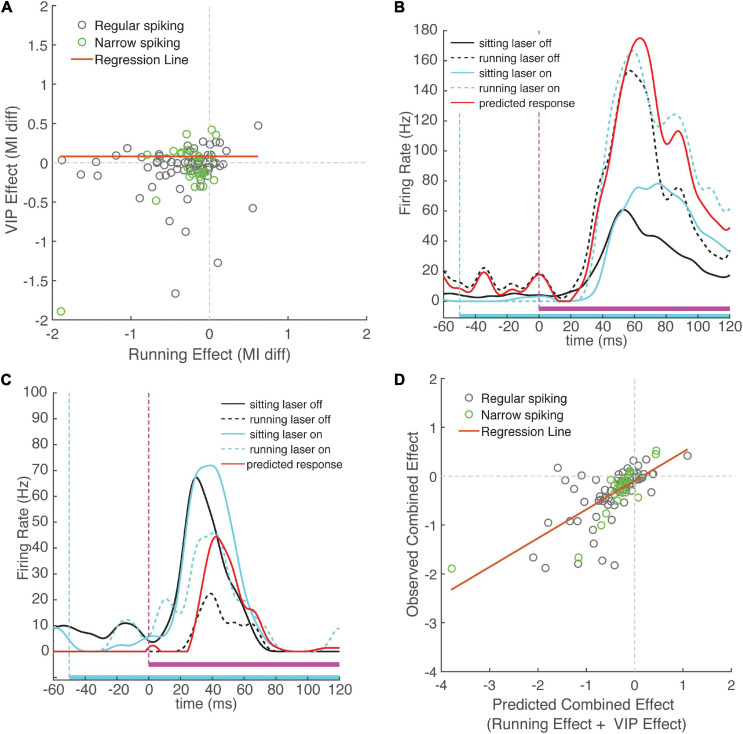
Change in sound modulation index during running laser-on trials is well-predicted by the sum of the running and VIP activation effects computed separately. **(A)** Running effect on sound modulation index plotted against VIP activation effect on sound modulation index for each neuron. The effects of running and activation VIP neurons were not correlated across the population of recorded cells (ρ = 0.11, *p* = 0.25). **(B)** Example neuron that exhibits an increase in activity during running and during VIP activation. Black traces show responses to WN during laser-off trials, cyan traces show WN responses show responses during laser-on trials. Mean responses during running trials are indicated with dashed lines. Red line indicates predicted combined effect of running and VIP activation (response sitting laser-off + change during running + change during VIP activation). Note the close match between the red line and the dashed blue line, indicating that the observed combined response closely matches that predicted by linear summation. **(C)** Example neuron showing suppression during running and facilitation during VIP activation. **(D)** Combined change in sound modulation during running and VIP activation plotted against predicted change in sound modulation index computed on running and VIP activation effect separately, showing strong correlation (ρ = 0.70, *p* < 0.001). Observed change in sound modulation during running laser-on trials can be well predicted by summing effects of running and VIP activation alone, suggesting that the effects of VIP activation and running do not interact.

If VIP neurons are a key mechanism underlying the modulatory effects of running, we would expect a predictive relationship between the effects of VIP activation and running. To test this idea, we compared the effect of VIP activation and running on sound MI in each recorded neuron (see section “Materials and Methods”) and found no correlation between these two modulatory effects (ρ = 0.11, *p* = 0.25, *N* = 99 cells, Figure 5A). Indeed, activation of VIP neurons explained only 1% of the variance in the cortical modulation produced by running. This suggests that VIP activation and modulation by running act through independent mechanisms.

To further investigate this idea, we examined the interaction between the two modulatory effects. By analogy with the concept of epistasis in genetics (Cordell, 2002), if running is not mediated by the VIP network, one would predict that the changes in cortical activity due only to running or only to VIP activation would sum linearly, since they are produced by independent mechanisms. In this case, the combined effect of both running and VIP activation on the same trial (running laser-on trials) would be strongly predicted by the arithmetic sum of the running effect (measured on running laser-off trials) and the VIP activation effect (measured on sitting laser-on trials). In contrast, if the running effect *is* mediated by the VIP network, we would expect a non-linear (sub-additive) interaction, because the effect of one manipulation precludes observing any additional effect of the other. We calculated expected effects for each neuron by summing the effects of running and VIP activation measured separately, and compared them to the observed change in sound MI during running laser-on trials. We found that the observed combined responses closely matched the expected combined response predicted by linear summation. Figure 5B shows an example of a cell with an onset response that was strongly facilitated by running and by VIP activation, showing the close match between the observed combined response and the expected combined response from linear summation. Figure 5C shows a cell that was suppressed by running and facilitated by VIP activation, again with a tight match between the observed and expected combined effects. Across cells, the observed combined effects and expected combined effects were highly correlated with one another (Figure 5D, ρ = 0.70, *p* = 10^–16^, *N* = 99), providing strong evidence for independent mechanisms for the two effects. Moreover, the expected combined effect values explained 43% of variance in the observed combined effect values (slope = 0.59, intercept = −0.09, *r*^2^ = 0.49, *p* = 10^–16^). To confirm that combined effect values were indeed better predictors than running effect or VIP effect values alone, we compared the performance of a linear regression model using the expected combined effect as a predictor to linear regression models using running and VIP effects as separate predictors. This analysis revealed that even though the running effect and the VIP effect are each predictive of the observed combined effect, their individual predictive power is lower than that when they are combined (running effect, *r*^2^ = 0.34, *p* = 10^–10^; VIP effect, *r*^2^ = 0.27, *p* = 10^–7^).

To confirm that the linear additivity we observed did not depend on the choice of response normalization (i.e., our use of sound MI), we repeated the analysis using non-normalized evoked and spontaneous firing rates separately. The changes in both evoked and spontaneous firing rates during running laser-on trials were well-predicted by the sum of firing rate changes during either running or laser-on trials (evoked: ρ = 0.81, *p* = 10^–37^, spontaneous: ρ = 0.92, *p* = 10^–56^, Supplementary Figure 6). This was true for both regular and narrow-spiking neurons (evoked: RS ρ = 0.76, NS ρ = 0.92; spontaneous: RS ρ = 0.90, NS ρ = 0.93).

## Discussion

It is now clear that locomotion has prominent effects in sensory cortex, and that the net effects in auditory cortex are the opposite of those in visual cortex. Are the mechanisms underlying these running effects similar or different in the two areas? In visual cortex, the VIP disinhibitory circuit facilitates evoked responses during running. Here we asked whether VIP neurons contribute to the running effect in auditory cortex. We found that both running and optogenetic activation of VIP neurons produced diverse changes in the firing rates of auditory cortical neurons, but with distinct effects on spontaneous and evoked activity and different patterns across cortical layers. On average, running increased spontaneous firing rates but decreased evoked firing rates, resulting in a reduction of the neuronal encoding of sound. This reduction in sound encoding was observed in all cortical layers, but was most pronounced in layer 2/3. In contrast, VIP activation increased both spontaneous and evoked firing rates, and had no net population-wide effect on sound encoding, but strongly suppressed sound encoding in layer 4 narrow-spiking neurons. Running and VIP activation also had opposing effects on response latency (an increase for running, but a decrease for VIP activation). These results suggest that VIP activation and running act independently, which we then tested by comparing the arithmetic sum of the two effects measured separately to the actual combined effect of running and VI P activation, which were closely matched. We conclude that the effects of locomotion in auditory cortex are not mediated by the VIP network. Our results do not rule out the possibility that locomotion acts in part through VIP cells while simultaneously engaging other pathways, such as projections from motor cortex, that together produce heterogenous effects in auditory cortex.

Our results confirm and extend recent findings that VIP activation increases firing rates in auditory cortex without any corresponding increase in the amount of stimulus information conveyed (Bigelow et al., 2019). We reached the same conclusion as that study, that movement effects and VIP activation act through independent pathways. Moreover, by recording with laminar precision we found that running effects were strongest in layer 2/3, whereas VIP effects were strongest in layer 4 and were driven primarily by suppression of sound-evoked responses in narrow-spiking cells. The mechanisms underlying the layer-specific effects of VIP activation remain an open question. One possibility is that direct inhibition of PNs differs across layers; another possibility is differential connectivity of the SOM neurons that are disinhibited by VIP activation. Although VIP neurons provide strong inhibition to SOM cells in L2/3, previous work has shown that their main GABAergic targets are interneurons in layers 5 and 6 (Zhou et al., 2017). Layer 4 contains a distinct class of non-Martinotti SOM cells, which do not target PNs (unlike Martinotti SOM cells in L2/3 and L5/6) and instead target narrow-spiking PV cells (Yavorska and Wehr, 2016). These L4 non-Martinotti SOM cells have narrow spikes, and thus could be included in our population of L4 NS cells. Our finding that evoked responses in L4 NS cells were suppressed by VIP activation might therefore be explained by narrow-spiking non-Martinotti SOM cells in layer 4 that are directly inhibited by VIP neurons, and could also be explained by directly or indirectly suppressed narrow-spiking PV cells in layer 4. Although previous work has emphasized the disinhibitory effects of the VIP→SOM network (Lee et al., 2013; Pfeffer et al., 2013; Pi et al., 2013), SOM neurons in layer 4 also inhibit PV cells (Xu et al., 2013), and VIP cells make a small number of synapses onto PV cells (Pfeffer et al., 2013). Thus there are potentially 2 or even 3 distinct disinhibitory circuits in L4 (VIP→SOM, SOM→PV, and VIP→PV). Although further work is required to clarify how these disinhibitory circuits interact, it seems likely that they could produce a mixture of inhibitory and disinhibitory effects. Consistent with this, we found substantial diversity in the effects of VIP activation (see Figure 3), as have others (Bigelow et al., 2019). Indeed, we found that activating VIP cells strongly modulated spontaneous and evoked activity in both regular and narrow-spiking neurons, suggesting that VIP cells, although few in number, nevertheless have a powerful effect on cortical activity.

Our conclusion that VIP neurons do not mediate running effects in auditory cortex is also aligned with several recent findings. In auditory cortex, running reduces sound-evoked activity and stimulus encoding, whereas VIP activation increased firing rates without affecting encoding (Bigelow et al., 2019). In visual cortex, VIP neurons respond specifically to novel images, and are suppressed by familiar images, a pattern which is unrelated to an animal’s movements (Garrett et al., 2020). This suggests an alternative role for the VIP network, related to learning and long-term memory (Krabbe et al., 2018; Garrett et al., 2020). If this role extends to auditory cortex as well, VIP neurons may be involved in learning and memory for complex sounds such as vocalizations or other natural stimuli. In somatosensory cortex, application of the VIP peptide also produced diverse inhibitory, excitatory, or biphasic responses in neurons (Sessler et al., 1991). When combined with other neurotransmitters such as GABA or ACh, VIP enhanced their effects, suggesting that it can act as a modulator. In addition, silencing VIP neurons does not block the desynchronization of sensory cortical neurons by cholinergic projections from the basal forebrain (Chen et al., 2015). The picture that emerges from these findings is that VIP circuitry not only produces diverse effects across neurons and cortical areas, but that these circuits influence brain function and behavior in ways well beyond the effects of locomotion and arousal.

Changes in behavioral state, such as running or changes in arousal, produce diverse inhibitory and excitatory effects on cortical neurons. We found that running led to a widespread increase in spontaneous activity in both regular and fast spiking neurons while simultaneously suppressing sound evoked responses (Figure 2). This dichotomy of running effects was present throughout cortical layers, although it varied in its strength. What possible mechanisms could lead to these differential effects on spontaneous and evoked activity? Previous research has shown that running suppresses auditory cortical evoked and spontaneous activity through a projection from M2 onto PV interneurons (Nelson et al., 2013; Schneider et al., 2014). This suppression of spontaneous activity by running is inconsistent with the increase in spontaneous activity that we and others have observed (Bigelow et al., 2019). This difference might be explained by different definitions of running conditions: Nelson et al. (2013) and Schneider et al. (2014) analyzed firing rates triggered at movement onset, whereas our analysis and that of Bigelow et al. (2019) compared between trials categorized as either running or sitting. These methodological differences could be exacerbated by the high degree of heterogeneity across neurons that we and others have observed. In addition to the M2 pathway, running also activates basal forebrain projections that target both excitatory neurons and most inhibitory cell subtypes (Nelson and Mooney, 2016). Unlike the M2 pathway, activation of these projections leads to widespread increases in the firing rates of auditory neurons via nicotinic acetylcholine signaling. Because M2 and the basal forebrain are driven by different sets of inputs, these two pathways for running modulation likely provide different types of feedback to auditory cortex. Activity in basal forebrain has been associated with arousal, attention, and plasticity in auditory cortex (Metherate et al., 1990; Everitt and Robbins, 1997; Sarter et al., 2005; Hangya et al., 2015) whereas M2 is involved in movement-related planning (Eliades and Wang, 2003). The interaction of these two pathways remains unclear, although some data suggests that changes in behavioral state might have a biphasic effect on auditory neurons. Neurons in auditory cortex show a depolarizing effect at the beginning of heightened arousal, which is followed by a hyperpolarizing period (Shimaoka et al., 2018), whereas running led to an overall suppression of evoked responses. Such biphasic effects of arousal suggest that some of the differences reported across studies might be attributable to timing differences in the measurements of modulatory effects.

In visual cortex, SOM neurons in L2/3 pool activity from local L2/3 PNs, such that the strength of the inhibition they provide is proportional to the increase in activity of PNs. Stimuli that span a large portion of the visual field strongly recruit SOM neurons via horizontal PN axons, producing surround suppression (Adesnik et al., 2012). Similarly, in auditory cortex, SOM neurons mediate surround suppression by broadband stimuli, which are the auditory equivalent of large visual stimuli (Lakunina et al., 2020), or by tones far from best frequency (Kato et al., 2017). Because VIP neurons inhibit SOM neurons, we would expect VIP activation to suppress SOM neurons, disrupt surround suppression, and thereby increase the responses evoked by our white noise (broadband) stimuli. Indeed, we found that VIP activation increased evoked responses (Figure 3B), confirming this prediction, but we also found that VIP activation increased spontaneous activity (Figure 3A), such that there was no net effect on sound encoding (Figures 3D,E). In contrast, directly suppressing SOM cells in visual cortex had no effect on spontaneous activity (Adesnik et al., 2012). This suggests that the effects of VIP activation on spontaneous and evoked activity likely act through independent pathways, with opposing effects on sound encoding, such that the net effect of these pathways is to cancel each other out to leave sound encoding unaffected.

## Experimental Procedures

### Mice

All procedures were performed in accordance with National Institutes of Health guidelines, as approved by University of Oregon Institutional Animal Care and Use Committee. We recorded from offspring of a cross between a homozygous cre-dependent ChR2-eYFP line (Madisen et al., 2012), JAX Stock No. 012569, and a homozygous VIP-IRES-Cre line (Taniguchi et al., 2011), JAX No. 010908, *N* = 10 mice, as well as offspring of a cross between the ChR2-eYFP line and a homozygous PV-IRES-Cre line, JAX No. 008069, *N* = 6 mice. We used both male and female mice, age range 60–210 days.

### Surgery

Mice were anesthetized with isoflurane (1.25–2.0%). A headpost was secured to the skull and a mark was made on the skull over auditory cortex for a future craniotomy (AP: −2.9 mm, ML: 4.4 mm, relative to bregma). Mice were housed individually following the surgery and were allowed at least 5 days of post-operative recovery. On the day of recording, mice were anesthetized with isoflurane (1.25–2.0%), the head was clamped with the headpost, and a small craniotomy was made over auditory cortex (1 × 1 mm). The craniotomy was covered with a thin layer of agar and the animal was allowed to recover for at least an hour before recording.

### Electrophysiology

All electrophysiological recordings were performed using linear array silicon probes while the animal was awake and head-fixed on a styrofoam ball inside a double-walled acoustic isolation booth. The ball was mounted on an axle that allowed it to rotate forward or backward; rotation of the ball produced by locomotion of the animal was measured with an optical mouse. Neurons in auditory cortex were recorded with either a 32-channel silicon probe (25 μm spacing between sites, single 750 μm shank, Neuronexus A1x32-Poly2-5mm-50s-177) or a 64-channel probe (25 μm spacing between sites, two 750 μm shanks, Neuronexus A2x32-Poly2-5mm-25s-200-177), Intan RHD2000 board, and Open Ephys software (Siegle et al., 2017). The silicon probe was positioned with a micromanipulator (Sutter MP-285) orthogonal to the cortical surface such that the electrode sites spanned cortical layers. Spiking and local field potential (LFP) data were filtered online (600–6000 Hz and 0.1–400 Hz, respectively) and recorded. Single neurons were identified offline using Kilosort spike sorting software (Pachitariu et al., 2016).

### Laminar Boundaries

To measure the depth of recorded cells, we used current source density analysis of the LFP evoked by 600 ms, 80 dB white noise bursts. We identified the robust short-latency sink at the L3–L4 boundary and assigned it a depth of 400 μm, and confirmed the presence of a source in L1 (Intskirveli and Metherate, 2012). We assigned the depths of individual neurons relative to this, based on the channel exhibiting the maximum waveform amplitude for each neuron. This allowed us to relate recording depth to our histological analysis and laminar boundaries (Anderson et al., 2009; Intskirveli and Metherate, 2012). Laminar boundaries were defined as follows: layer 1 = 0–128 μm, layer 2/3 = 129–380 μm, layer 4 = 381–525 μm, layer 5 = 526–805 μm, layer 6 = 806–1200 μm (Weible et al., 2020). Only one neuron was classified as a layer 1 neuron. Recordings for which current-source density did not yield unambiguous depth information were excluded from any analysis based on depth of recorded cells (i.e., laminar analysis). For the running/sitting analyses (Figure 2), we kept 250/524 cells from recordings with clear laminar current source density (CSD) patterns, and excluded the remainder from laminar analysis. For the VIP activation analysis (Figure 4), we kept 648/1009 cells and excluded the remainder from laminar analysis. Because the recording sites on our probe only spanned 750 μm, layer 6 was relatively undersampled in our dataset.

### Optogenetic Illumination

An optic fiber (200 μm diameter) was attached to the silicon probe such that the tip of the fiber was 1500 μm away from the tip of the probe, allowing recording from the full cortical column while not directly touching the cortical tissue. Blue light (450 μm) was delivered from a diode laser using a 200 μm diameter optic fiber. The light power was calibrated at the beginning of each experiment to 10 mW (corresponding to 317 mW/mm^2^ at the tip of the fiber). Timing of laser pulses is described below.

### Acoustic Stimuli

Sound was delivered from a free field speaker contralateral to the recording hemisphere. To test effects of locomotion and VIP activation on evoked activity, we presented 600 ms white noise (WN) bursts at 80 dB amplitude with a 1 s interstimulus interval. Acoustic stimuli were randomly interleaved with a blank stimulus (a 600 ms period of silence), used for measuring spontaneous activity. Both white noise and silent stimuli presentations were presented with and without laser illumination, randomly interleaved (See Figure 1A for experimental design). The laser pulses were 800 ms in duration and began 50 ms before the start of the sound and ended 150 ms after sound offset. During each pulse, the laser was continuously on (DC illumination). Stimuli were presented at least 30 times in each combination (WN, blank, WN + laser, blank + laser).

### Behavioral State Recording

Running speed was recorded using pulse width modulation via an optic mouse that was connected to a Raspberry Pi. Movements of the ball were detected by the optic mouse which modulated the width of a 10 ms pulse that the Raspberry Pi sent to the Intan board. We smoothed the running trace with a 200 ms window moving median average.

### Analysis

#### Modulation Index

We computed firing rates by binning spike times of individual neurons in 5 ms windows. Onset responses and offset responses were quantified as the average firing rate (FR) in 0–100 ms and 600–700 ms time windows relative to stimulus onset, respectively. Significant evoked responses were identified by a Wilcoxon rank-sum test between neuronal firing rate during sound presentation trials and spontaneous neural firing rate during a matched period of silence on laser-off trials (*p* < 0.01). Analysis of the evoked responses included all cells that responded significantly to sound with an increase in firing rate. MI was defined as the difference between the mean evoked firing rate when the sound was presented and the mean spontaneous firing rate, divided by the sum of those means:

$$\begin{array}{c} SoundMIsittinglaser\text{-}off=\\ \frac{\left(meanEvokedFR_{sitlaser\text{-}off}-meanSpontFR_{sitlaser\text{-}off}\right)}{\left(meanEvokedFR_{sitlaser\text{-}off}+meanSpontFR_{sitlaser\text{-}off}\right)} \end{array}$$

$$\begin{array}{c} SoundMIrunninglaser\text{-}off=\\ \frac{\left(meanEvokedFR_{runlaser\text{-}off}-meanSpontFR_{runlaser\text{-}off}\right)}{\left(meanEvokedFR_{runlaser\text{-}off}+meanSpontFR_{runlaser\text{-}off}\right)} \end{array}$$

$$\begin{array}{c} SoundMIsittinglaser\text{-}on=\\ \frac{\left(meanEvokedFR_{sitlaser\text{-}on}-meanSpontFR_{sitlaser\text{-}on}\right)}{\left(meanEvokedFR_{sitlaser\text{-}on}+meanSpontFR_{sitlaser\text{-}on}\right)} \end{array}$$

$$\begin{array}{c} SoundMIrunninglaser\text{-}on=\\ \frac{\left(meanEvokedFR_{runlaser\text{-}on}-meanSpontFR_{runlaser\text{-}on}\right)}{\left(meanEvokedFR_{runlaser\text{-}on}+meanSpontFR_{runlaser\text{-}on}\right)} \end{array}$$

Because we included only increased responses to sound (on sitting laser-off trials), the sound MI varies from 0 to 1 in laser-off sitting trials, and measures the amount of firing rate modulation induced by sound presentation. For neurons that were suppressed by sound during laser-on or running trials, MI could decrease as low as −1. For example, a neuron with a sound MI_*sitting*_ close to 1 and sound MI_*running*_ close to 0 responded much more to sound when the mouse was sitting still. A neuron with a sound MI_*running*_ of −1 had an evoked response that was completely suppressed during running. We computed each MI during different conditions; for example, Sound MI_*sitting laser–on*_ computes modulation by sound during VIP activation trials (i.e., laser-on), recorded when the mouse was sitting still.

#### Interaction Analysis

To obtain predicted values for each neuron, we first computed sound MI for each neuron in four separate conditions: sitting laser-off, running laser-off, sitting laser-on, and running laser-on (see section “Modulation Index”). To calculate the effect of running, we took the difference between Sound MI_*running laser–off*_ and Sound MI_*sitting laser–off*_ (range: −2 to 2).

$$\begin{array}{c} Running\;Effect=\\ Sound\;MI_{running\;laser\text{-}off}-Sound\;MI_{sitting\;laser\text{-}off} \end{array}$$

To calculate the effect of VIP activation, we took the difference in Sound MI_*sitting laser–on*_ condition and Sound MI_*sitting laser–off*_ condition (range: −2 to 2).

$$\begin{array}{c} VIP\;Effect=\\ Sound\;MI_{sitting\;laser\text{-}on}-Sound\;MI_{sitting\;laser\text{-}off} \end{array}$$

The predicted value for each cell was the sum of the effects of behavioral modulation (running) and VIP activation (laser-on), range: −4 to 4.

$$\begin{array}{c} Predicted\;Combined\;Effect=\\ Running\;Effect_{laser\text{-}off}+VIP\;Effect_{sit} \end{array}$$

To measure the actual modulation when both experimental conditions were present simultaneously (running + VIP activation), we computed a new MI for each cell, Sound MI_*running laser–on*_ (see section “Modulation Index”). Because we included only neurons that had sufficient number of running and laser trials, this resulted in a smaller subset of cells (*N* = 99).

$$\begin{array}{c} Observed\;Combined\;effect=\\ Sound\;MI_{running\;laser\text{-}on}-Sound\;MI_{sitting\;laser\text{-}off} \end{array}$$

#### Current Source Densities

Current source densities were computed from LFPs recorded during presentation of acoustic stimuli (white noise at 80 dB). LFPs were bandpass filtered from 1 to 300 Hz to remove spikes. CSDs were computed using the standard method; i.e., the second spatial derivative of LFPs were estimated by the corresponding spatial differences (Pettersen et al., 2006).

$$\begin{array}{c} CSD_{j}=\\ \left(trace_{j-1}+trace_{j+1}-2^{*}trace_{j}\right)/distance^{2} \end{array}$$

This resulted in identifiable evoked sources and sinks with characteristic spatiotemporal patterns across the laminar structure of auditory cortex (Intskirveli and Metherate, 2012).

#### Cell Type Categorization

To categorize cells as narrow-spiking or regular-spiking we measured spike width, end-slope, and peak-to-trough ratio of spike waveforms in recorded neurons. Width was measured as a distance from the peak to the trough of spike waveform. Neurons showed a clear separation into two clusters based on their spike width, thus cells that had a spike width of less than 0.5 ms and negative end-slope were classified as narrow-spiking cells (NS), while cells that had spike with of 0.5 ms or greater were classified into regular-spiking (RS) cells (Niell and Stryker, 2008; Moore and Wehr, 2013). RS and NS cells differed in their spontaneous (*p* = 0.0155) and evoked firing rates (*p* = 10^–5^, *N* = 372).

#### VIP Activation Experiments

We recorded from 10 VIP-ChR2 mice, *N* = 27 recording sessions, *N* = 1009 total cells, regular-spiking: *N* = 834, narrow-spiking: *N* = 175. For a subset of recorded neurons (*N* = 648), we were able to assign the cortical layer they belonged to by identifying sources and sinks evoked by a white noise stimulus using current source density analysis. Modulation induced by running was calculated for a subset of recordings in which we were able to obtain a sufficient number of trials in each condition (*N* = 11 recording sessions). Although we attempted to optogenetically identify VIP+ neurons based on their direct responses to light, we could not unambiguously identify enough cells to include in this report.

#### Distance Correlation

Distance correlation values were computed between binned firing rates of simultaneously recorded neurons (100 ms bins) and running speed (Székely et al., 2007). Because distance correlation measures both linear and non-linear relationships between two variables, the values of the relationship between two variables can be only positive or zero. A distance correlation value of zero can be obtained only if there is no observed dependency between two variables. Additionally, distance correlation can be computed between two variables of different dimensions. Neuronal activity was defined by an *n* by *t* matrix where *n* is the number of neurons in the recording and *t* is the duration of the recording. Running speed was defined by an array of length *t*, the duration of recording. We first computed distance correlations in time bins ranging from 50 ms to 40 s, to test the dependence between neural activity and running at different timescales. We then computed distance correlations using the same time bins but randomly shuffling the running trace as a shuffle control. We then subtracted the shuffle control from the measured distance correlation to obtain an estimate of the relationship between running and neural activity at different time scales. To calculate significance of distance correlation values, we repeated the analysis 50 times with a newly shuffled running trace each time. Significance was obtained by dividing the number of times the shuffled trace led to a distance correlation value that was equal or higher than a true distance correlation from non-shuffled data by the total number of trials.

#### Response Latency

We computed response latency for each cell by smoothing the trial-averaged firing rate with a 15-ms sliding window average, and finding the time point of half-maximal firing rate (halfway between 0 Hz and the peak firing rate).

#### Statistics

All statistical analysis was performed in Matlab. We used non-parametric statistical tests because not all data were normally distributed. We used the two-sided Wilcoxon signed-rank test to compare within-group effects (e.g., change in firing rate or MI in running vs. sitting condition, Figures 2A–C, 3A–C), and the two-sided Wilcoxon rank-sum test to compare between-group effects (e.g., firing rates and modulation indices of RS vs. NS cells). For effect sizes we used *r* (Rosenthal et al., 1994), calculated as

$$r=z/\sqrt{N}$$

where z is the Wilcoxon test statistic and *N* is the total number of cases (i.e., twice the sample size for within-group comparisons). For comparison of multiple groups (e.g., cortical layers, Figures 2E, 4), we tested for group differences using the Kruskal–Wallis test, followed by rank-sum *post-hoc* tests using a Bonferroni multiple comparisons correction. Unless stated otherwise, data are reported as group means and standard error of the mean (mean ± SEM).

#### Within-Animal Correlations

Because we recorded from a large sample size of neurons from a comparatively smaller number of recording sessions and mice, we checked to make sure our results were not skewed by within-animal correlations. The effect of running was not different across recordings (Kruskal–Wallis χ^2^ = 7.3, *p* = 0.75, *N* = 12 recording sessions) or across mice (χ^2^ = 6.13, *p* = 0.53, *N* = 8 mice). This shows that the effects of running we observed are robust across recordings and animals. The effect of VIP activation showed greater variability, with a significant difference across recordings (χ^2^ = 43.72, *p* = 0.01, *N* = 27 recording sessions) and across mice (χ^2^ = 20.48, *p* = 0.001, *N* = 8 mice). These differences were driven by only two recordings that were significantly different from one another, and by one mouse that was significantly different from two other mice. Excluding the two recordings that were different from one another did not change any of the results presented in this report. This shows that the effects of VIP activation are also robust across recordings and animals.

## Data Availability Statement

The data that support the findings of this study are available at http://www.uoneuro.uoregon.edu/wehr/data.html.

## Ethics Statement

The animal study was reviewed and approved by University of Oregon Institutional Animal Care and Use Committee.

## Author Contributions

IY performed the research and data analysis. Both authors designed the research and wrote the manuscript.

## Conflict of Interest

The authors declare that the research was conducted in the absence of any commercial or financial relationships that could be construed as a potential conflict of interest.

## Supplementary Material

Supplementary Figure 1 Running occurred during periods of high arousal, as measured by pupil diameter. Curves show the probability distribution of recorded pupil diameters (normalized to the maximum diameter in each recording session), separately for sitting (red) and running (black).

Supplementary Figure 2 Offset responses showed similar modulation by running as onset responses, suggesting running has general effects across multiple aspects of sound processing. Offset responses showed a modest but significant decrease during running. **(A)** Offset response firing rate evoked by white noise stimulus (100 ms window following stimulus offset) during sitting and running trials (without baseline subtraction). Red filled circle: population mean, red unfilled circle: median. Dashed line is unity. Mean evoked offset responses: running 12.06 ± 0.78 Hz, sitting 12.85 ± 0.78 Hz, signed-rank *p* = 0.0102, *N* = 206 cells, effect size *r* = 0.13. **(B)** Spontaneous firing rate during sitting and running trials. Running increased spontaneous firing rates. Green: narrow-spiking neurons, grey: regular-spiking neurons. These data are similar to those in Figure 2A, but not identical, because these are the subset of cells with significant offset responses (whereas the cells in Figure 2A were those with significant onset responses). **(C)** Offset response sound modulation index during sitting trials plotted against sound modulation index during running trials. Modulation index was strongly suppressed by running (*p* = 0.0102, effect size *r* = 0.13), because evoked firing rates were reduced while spontaneous firing rates were increased. **(D)** Distributions of offset response sound modulation indices during sitting (solid line) and running (dashed line). **(E)** Mean offset response sound modulation indices during sitting and running. **(F)** Mean and SEM of offset response sound modulation indices across cortical layers in sitting and running conditions (L2/3 sitting = 00.48 ± 0.03, running = 0.27 ± 0.05, *N* = 12; L4 sitting = 0.43 ± 0.02, running = 0.12 ± 0.03, *N* = 27; L5 sitting = 0.40 ± 0.01, running = 0.19 ± 0.02, *N* = 62; L6 sitting = 0.53 ± 0.03, running = 0.19 ± 0.05, *N* = 14; χ^2^ (3, 111) = 4.5, *p* = 0.21).

Supplementary Figure 3 Running increased response latency, whereas VIP activation decreased response latency. **(A)** Response to a white noise onset, averaged across either RS or NS cells, in running and sitting conditions (laser off). Running significantly increased response latency in both RS cells (*p* = 0.014, sign-rank, latency difference: 4.9 ± 1.2 ms, *r* = 0.17) and FS cells (*p* = 0.014, latency difference: 9.3 ± 1.7 ms, *r* = 0.27). **(B)** Response to a white noise onset, averaged across either RS or NS cells, in either laser-on or laser-off conditions (during sitting). VIP activation modestly but significantly decreased response latency in both RS cells (*p* = 0.02, sign-rank, latency difference: −2.4 ± 0.8 ms, *r* = −0.23) or FS cells (*p* = 0.07, latency difference: −4.7 ± 1.3 ms, *r* = −0.21).

Supplementary Figure 4 Effects of VIP activation on Sound MI for RS and NS cells. Mean sound modulation index during laser-on and laser-off trials, across cortical layers (same as Figure 4A), here plotted separately for RS and NS cells. VIP activation suppressed modulation of neural activity by sound in layer 4 NS cells, but not in RS cells or in NS cells in other layers. L2/3 *N* = 10 RS cells, *N* = 10 NS cells; L4 *N* = 28 RS cells, *N* = 12 NS cells; L5 *N* = 126 RS cells, *N* = 52 NS cells; L6 *N* = 3 RS cells, *N* = 3 NS cells.

Supplementary Figure 5 Distance correlation between running and population activity confirms that running strongly modulates firing in auditory cortex. We measured the relationship between running speed and spontaneous activity during prolonged periods of silence, by computing the distance correlation jointly between running speed and the firing rates of all simultaneously recorded neurons. To test the timescale of this relationship, we binned firing rates into bins ranging from 50 ms to 12.8 s. Running speed was significantly correlated with population activity across all time bins, with a broad peak at 0.4–0.8 s. Thus running is correlated with auditory cortical activity at a time scale of about half a second. *N* = 67 simultaneously recorded populations in 12 mice.

Supplementary Figure 6 Linearity analysis with firing rate. To verify that the linear additivity we observed did not depend on the choice of response normalization (i.e., our use of sound modulation index), we repeated the analysis of Figure 5 using non-normalized evoked and spontaneous firing rates separately. The changes in both evoked and spontaneous firing rates during running laser-on trials were well-predicted by the sum of firing rate changes during running and laser-on trials. This was true for both regular and narrow-spiking neurons. **(A)** Change in evoked firing rate (FR change) during running laser-on trials was well-predicted by the sum of the running and VIP activation effects computed separately (Expected FR change), ρ = 0.81, *p* = 10^–37^, suggesting that the effects of VIP activation and running do not interact. Green: narrow-spiking neurons, grey: regular-spiking neurons. **(B)** Change in spontaneous firing rate (FR) during running laser-on trials was well-predicted by the sum of the running and VIP activation effects computed separately, ρ = 0.92, *p* = 10^–56^. **(C)** Running effects and VIP activation effects on evoked firing rates were weakly correlated across neurons. Running effect is on the *x*-axis (FR change on running laser-off trials), and VIP activation effect is on the *y*-axis (FR change on sitting laser-on trials), ρ = 0.2977, *p* = 0.004. **(D)** Running effects and VIP activation effects on spontaneous firing rates were not correlated across neurons, ρ = −0.0853, *p* = 0.38. **(E)** As an alternative method to verify the linear additivity we observed, we computed a modulation index for VIP activation: $VIPMI=\frac{laser-on-laser-off}{laser-on+laser-off}$, a modulation index for running: $runningMI=\frac{running-sitting}{running+sitting}$, and a modulation index for the combined effect of running during VIP activation: $VIP+runningMI=\frac{runninglaser-on-sittinglaser-off}{runninglaser-on+sittinglaser-off}$. We then compared the actual VIP+ running MI to the predicted sum of VIP MI and running MI for evoked firing rates, finding a tight correlation between observed and expected effects, ρ = 0.7478, *p* = 10^–19^. **(F)** Same analysis as in panel **(E)** but for spontaneous firing rates. Actual VIP+running MI was well predicted by the sum of VIP MI and running MI for spontaneous firing rates, ρ = 0.7543, *p* < 10^–20^. **(G)** An alternative method to verify that running effects and VIP activation effects were independent of one another. Comparison of VIP MI and running MI (defined above in panel **E**) for evoked firing rates showed the two were uncorrelated, ρ = 0.1068, *p* = 0.29. **(H)** Same analysis as in panel **(G)** but for spontaneous firing rates. VIP MI and running MI for spontaneous firing rates were uncorrelated, ρ = 0.1035, *p* = 0.20.
