# Supplementary material for: Effects of Locomotion in Auditory Cortex Are Not Mediated by the VIP Network
Source: Front Neural Circuits. 2021 Apr 7;15:618881. doi: 10.3389/fncir.2021.618881 (PMC8058405; doi:10.3389/fncir.2021.618881)
Supplement: Supplementary Figure 1 — Running occurred during periods of high arousal, as measured by pupil diameter. Curves show the probability distribution of recorded pupil diameters (normalized to the maximum diameter in each recording session), separately for sitting (red) and running (black). [file Data_Sheet_1.PDF]

Effects of locomotion in auditory cortex are not mediated by the VIP network

Iryna Yavorska and Michael Wehr

University of Oregon

Supplemental Figures

Supplemental Figure 1. Running occurred during periods of high arousal, as measured by pupil diameter.

Supplemental Figure 2. Offset responses showed similar modulation by running as onset responses, suggesting running has general effects across multiple aspects of sound processing. Offset responses showed a modest but significant decrease during running.

Supplemental Figure 3. Running increased response latency, whereas VIP activation decreased response latency.

Supplemental Figure 4. Effects of VIP activation on Sound MI for RS and NS cells
Mean sound modulation index during laser-on and laser-off trials, across cortical layers (same as Figure 4A), plotted separately for RS and NS cells.

Supplemental Figure 5. Distance correlation between running and population activity confirms that running strongly modulates firing in auditory cortex.

Supplemental Figure 6. Linearity Analysis with firing rate. To verify that the linear additivity we observed did not depend on the choice of response normalization (i.e., our use of sound modulation index), we repeated the analysis of Figure 5 using non-normalized evoked and spontaneous firing rates separately.

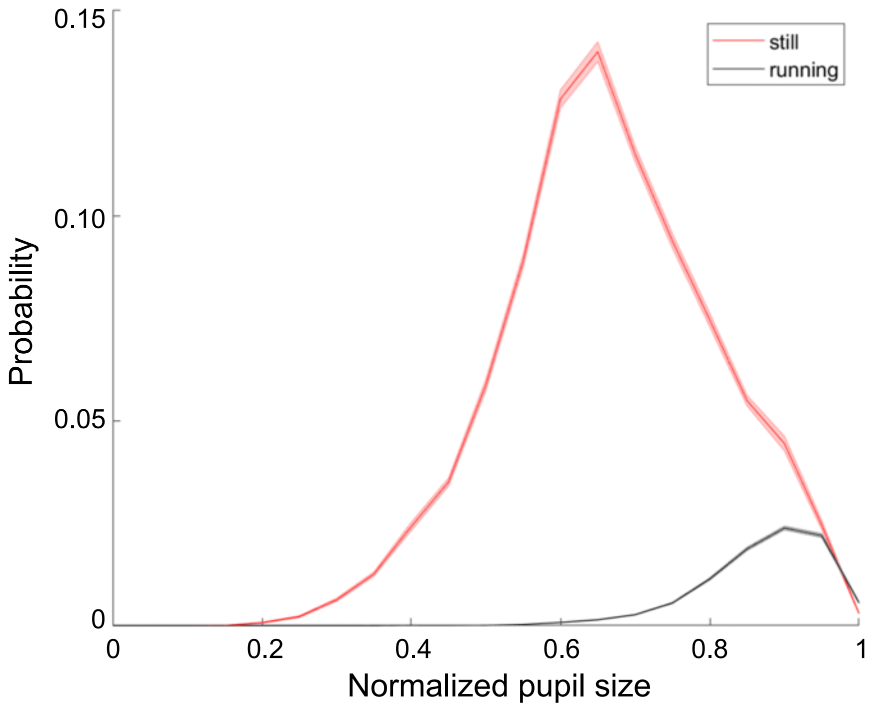

Supplemental Figure 1. Running occurred during periods of high arousal, as measured by pupil diameter. Curves show the probability distribution of recorded pupil diameters (normalized to the maximum diameter in each recording session), separately for sitting (red) and running (black).

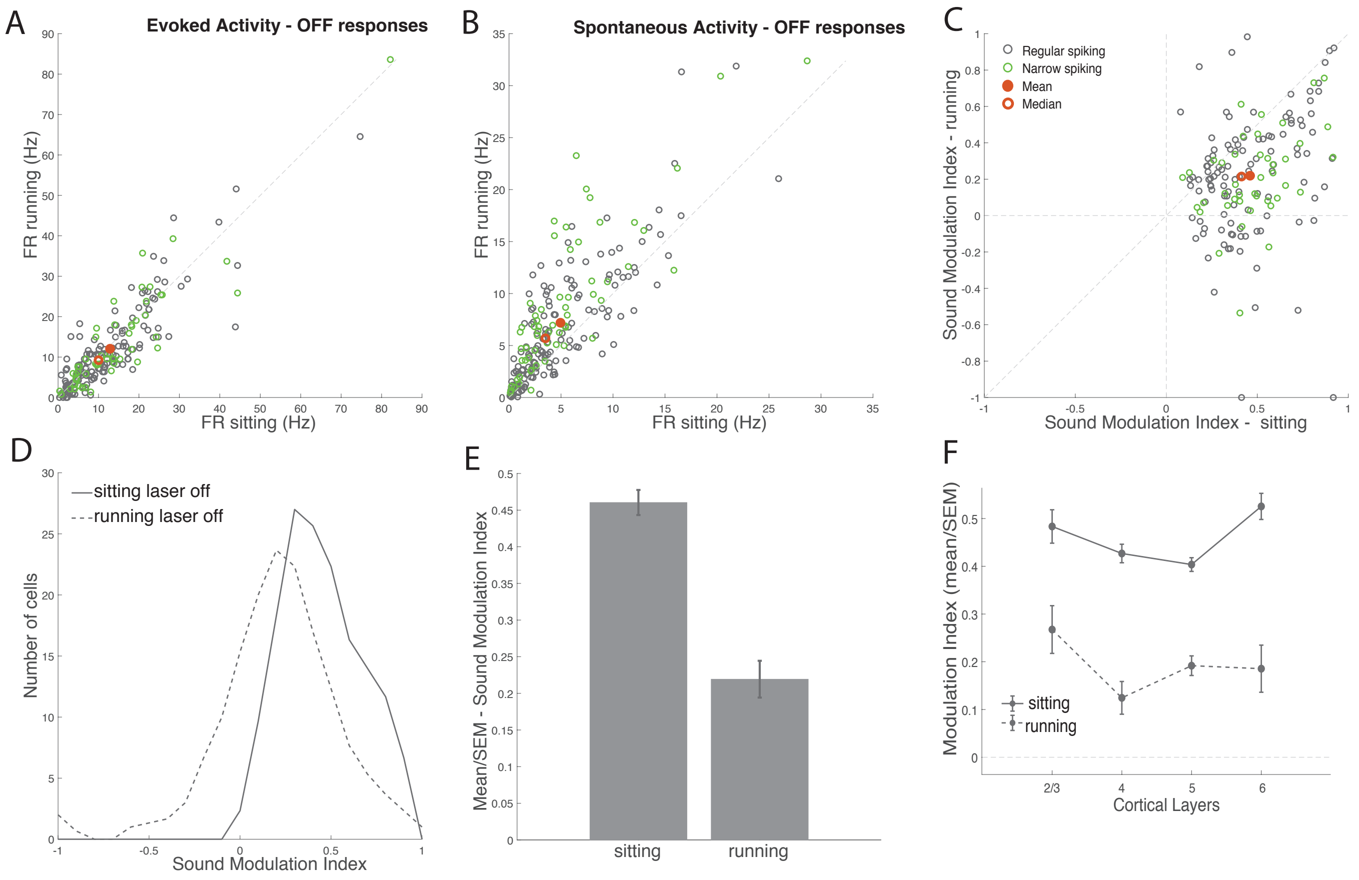

Supplemental Figure 2. Offset responses showed similar modulation by running as onset responses, suggesting running has general effects across multiple aspects of sound processing. Offset responses showed a modest but significant decrease during running.

A. Offset response firing rate evoked by white noise stimulus (100 ms window following stimulus offset) during sitting and running trials (without baseline subtraction). Red filled circle: population mean, red unfilled circle: median. Dashed line is unity. Mean evoked offset responses: running 12.06 ± 0.78 Hz, sitting 12.85 ± 0.78 Hz, signed-rank p = 0.0102, N = 206 cells, effect size r = 0.13.

B. Spontaneous firing rate during sitting and running trials. Running increased spontaneous firing rates. Green: narrow-spiking neurons, grey: regular-spiking neurons. These data are similar to those in Fig. 2A, but not identical, because these are the subset of cells with significant offset responses (whereas the cells in Fig. 2A were those with significant onset responses).

C. Offset response sound modulation index during sitting trials plotted against sound modulation index during running trials. Modulation index was strongly suppressed by running (p = 0.0102, effect size r = 0.13), because evoked firing rates were reduced while spontaneous firing rates were increased.

D. Distributions of offset response sound modulation indices during sitting (solid line) and running (dashed line).

E. Mean offset response sound modulation indices during sitting and running.

F. Mean and SEM of offset response sound modulation indices across cortical layers in sitting and running conditions (L2/3 sitting = 00.48 ± 0.03, running = 0.27 ± 0.05, n = 12; L4 sitting = 0.43 ± 0.02, running = 0.12 ± 0.03, n = 27; L5 sitting = 0.40 ± 0.01, running = 0.19 ± 0.02, = 62; L6 sitting = 0.53 ± 0.03, running = 0.19 ± 0.05, n = 14; $\mathcal{X}^2$ (3, 111) = 4.5, p = 0.21)

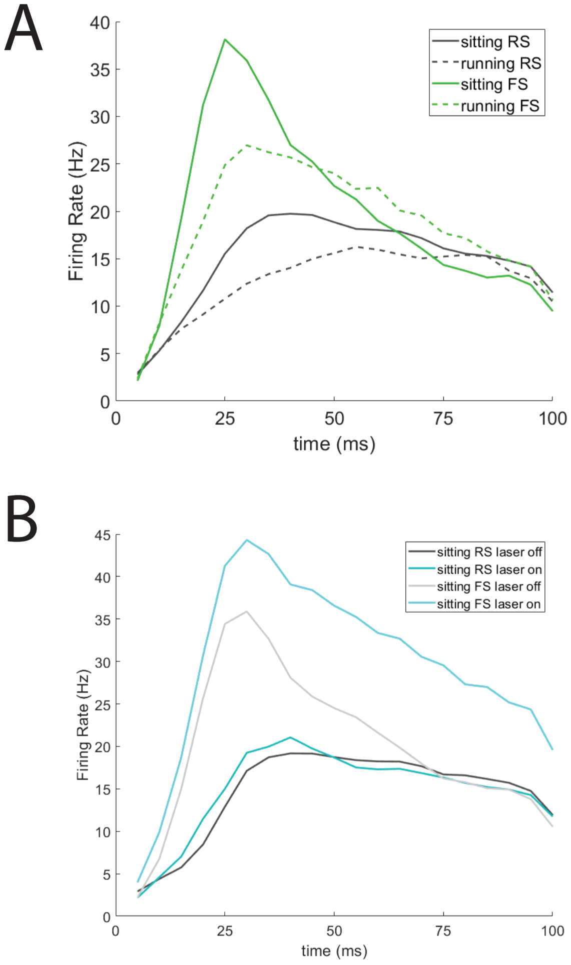

Supplemental Figure 3. Running increased response latency, whereas VIP activation decreased response latency.

A. Response to a white noise onset, averaged across either RS or NS cells, in running and sitting conditions (laser off). Running significantly increased response latency in both RS cells ($p = 0.014$, sign-rank, latency difference: 4.9 ± 1.2 ms, $r = 0.17$) and FS cells ($p = 0.014$, latency difference: 9.3 ± 1.7 ms, $r = 0.27$).

B. Response to a white noise onset, averaged across either RS or NS cells, in either laser-on or laser-off conditions (during sitting). VIP activation modestly but significantly decreased response latency in both RS cells ($p = 0.02$, sign-rank, latency difference: -2.4 ± 0.8 ms, $r = -0.23$) or FS cells ($p = 0.07$, latency difference: -4.7 ± 1.3 ms, $r = -0.21$).

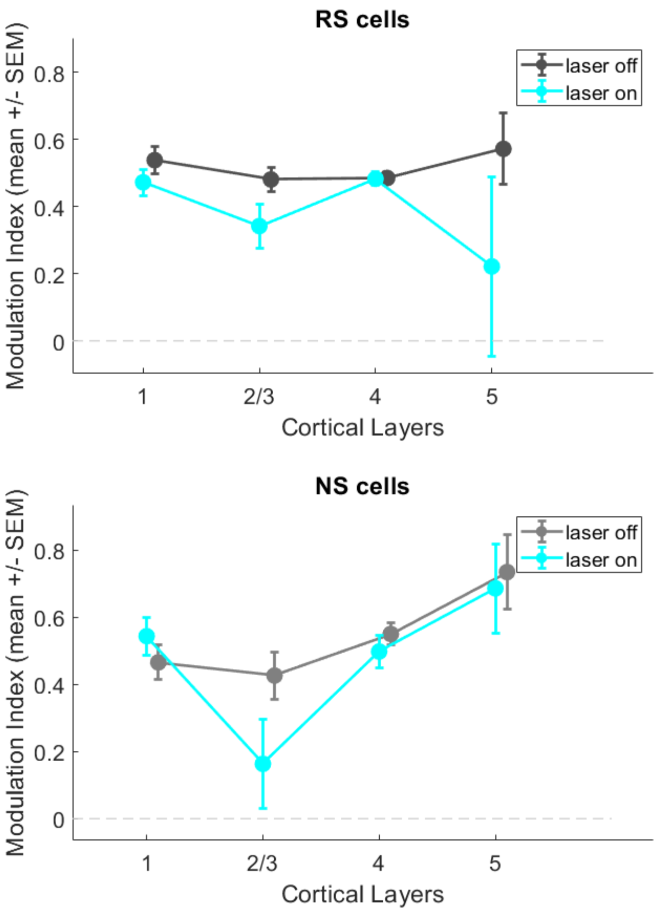

Supplemental Figure 4. Effects of VIP activation on Sound MI for RS and NS cells
Mean sound modulation index during laser-on and laser-off trials, across cortical layers (same as Figure 4A), here plotted separately for RS and NS cells. VIP activation suppressed modulation of neural activity by sound in layer 4 NS cells, but not in RS cells or in NS cells other layers. L2/3 N = 10 RS cells, N = 10 NS cells; L4 N = 28 RS cells, N = 12 NS cells; L5 N = 126 RS cells, N = 52 NS cells; L6 N = 3 RS cells, N = 3 NS cells.

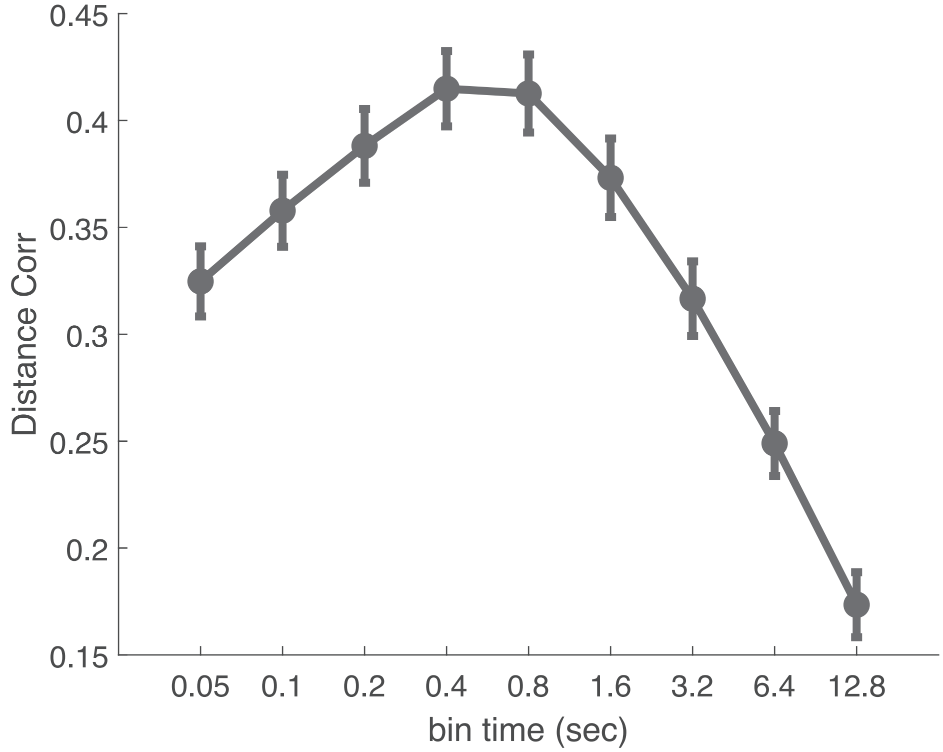

Supplemental Figure 5. Distance correlation between running and population activity confirms that running strongly modulates firing in auditory cortex. We measured the relationship between running speed and spontaneous activity during prolonged periods of silence, by computing the distance correlation jointly between running speed and the firing rates of all simultaneously recorded neurons. To test the timescale of this relationship, we binned firing rates into bins ranging from 50 ms to 12.8 s. Running speed was significantly correlated with population activity across all time bins, with a broad peak at 0.4 s. Thus running is correlated with auditory cortical activity at a time scale of a few hundred miliseconds. N = 67 simultaneously recorded populations in 12 mice.

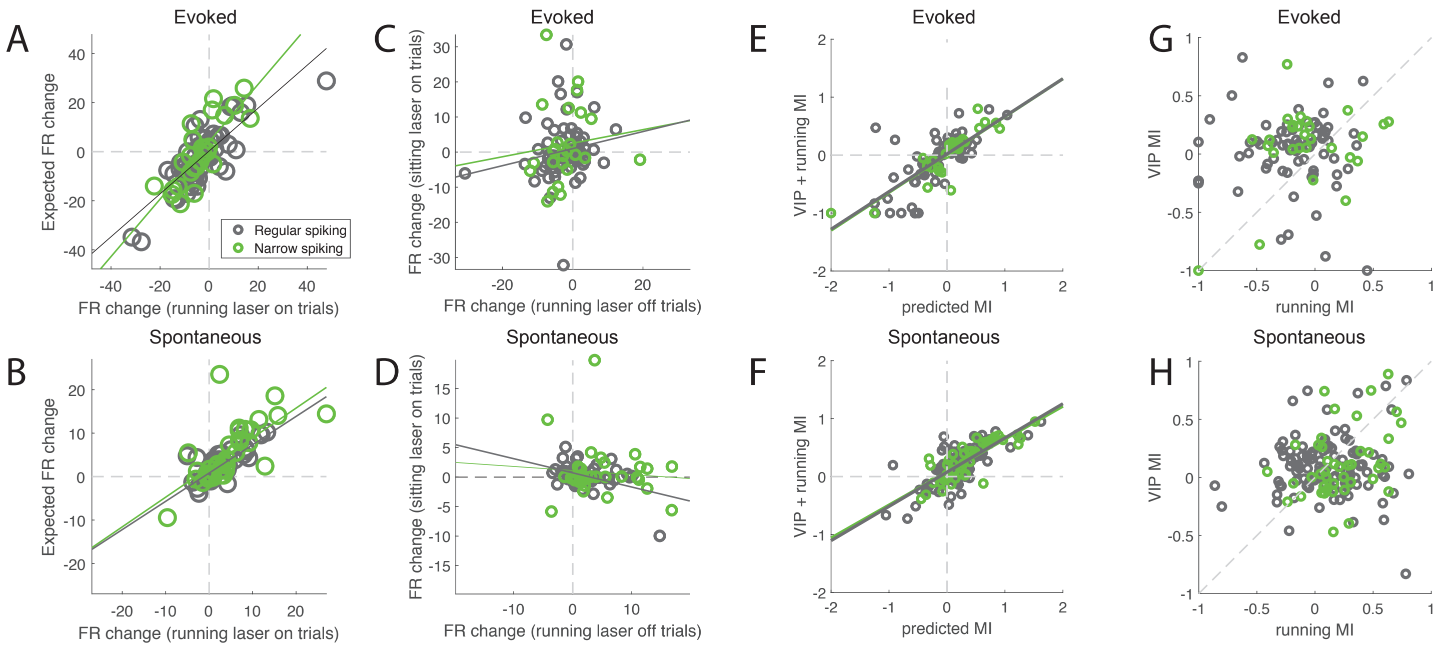

Supplemental Figure 6. Linearity Analysis with firing rate. To verify that the linear additivity we observed did not depend on the choice of response normalization (i.e., our use of sound modulation index), we repeated the analysis of Figure 5 using non-normalized evoked and spontaneous firing rates separately. The changes in both evoked and spontaneous firing rates during running laser-on trials were well-predicted by the sum of firing rate changes during either running or laser-on trials. This was true for both regular and narrow-spiking neurons

A. Change in evoked firing rate (FR change) during running laser-on trials was well-predicted by the sum of the running and VIP activation effects computed separately (Expected FR change), $\rho = 0.81$, $p = 10^{-37}$, suggesting that the effects of VIP activation and running do not interact. Green: narrow-spiking neurons, grey: regular-spiking neurons.

B. Change in spontaneous firing rate (FR) during running laser-on trials was well-predicted by the sum of the running and VIP activation effects computed separately, $\rho = 0.92$, $p = 10^{-56}$.

C. Running effects and VIP activation effects on evoked firing rates were weakly correlated across neurons. Running effect is on the x-axis (FR change on running laser-off trials), and VIP activation effect is on the y-axis (FR change on sitting laser-on trials), $\rho = 0.2977$, $p = 0.004$.

D. Running effects and VIP activation effects on spontaneous firing rates were not correlated across neurons, $\rho = -0.0853$, $p = 0.38$.

E. As an alternative method to verify the linear additivity we observed, we computed a modulation index for VIP activation: $VIP\ MI = \frac{laser-on\ -\ laser-off}{laser-on\ +\ laser-off}$, a modulation index for running: $running\ MI = \frac{running\ -\ sitting}{running\ +\ sitting}$, and a modulation index for the combined effect of running during VIP activation: $VIP + running\ MI = \frac{running\ laser-on\ -\ sitting\ laser-off}{running\ laser-on\ +\ sitting\ laser-off}$. We then compared the actual VIP+running MI to the predicted sum of VIP MI and running MI for evoked firing rates, finding a tight correlation between observed and expected effects, $\rho = 0.7478$, $p = 10^{-19}$.

F. Same analysis as in (E) but for spontaneous firing rates. Actual VIP+running MI was well predicted by the sum of VIP MI and running MI for evoked firing rates, $\rho = 0.7543$, $p < 10^{-20}$.

G. An alternative method to verify that running effects and VIP activation effects were independent of one another. Comparison of VIP MI and running MI (defined above in E) for evoked firing rates showed the two were uncorrelated, $\rho = 0.1068$, $p = 0.29$.

H. Same analysis as in (G) but for spontaneous firing rates. VIP MI and running MI for spontaneous firing rates were uncorrelated, $\rho = 0.1035$, $p = 0.20$.
